# Atomic-Resolution Three-Dimensional Structure of Amyloid β Fibrils Bearing the Osaka Mutation**

**DOI:** 10.1002/anie.201408598

**Published:** 2014-11-13

**Authors:** Anne K Schütz, Toni Vagt, Matthias Huber, Oxana Y Ovchinnikova, Riccardo Cadalbert, Joseph Wall, Peter Güntert, Anja Böckmann, Rudi Glockshuber, Beat H Meier

**Affiliations:** Physical Chemistry, ETH Zürich, Vladimir-Prelog-Weg 28093 Zurich (Switzerland); Institute of Molecular Biology and Biophysics, ETH ZurichOtto-Stern-Weg 5, 8093 Zurich (Switzerland); Brookhaven National Laboratory, 50 Bell AvenueBuilding 463,Upton, NY 11973-5000 (USA); Institute of Biophysical ChemistryCenter for Biomolecular Magnetic Resonance and Frankfurt Institute for Advanced Studies, Goethe University, Frankfurt am Main (Germany); Institut de Biologie et Chimie des ProtéinesBases Moléculaires et Structurales des Systèmes Infectieux, Labex Ecofect, UMR 5086 CNRS, Université de Lyon, 7 passage du Vercors, 69367 Lyon (France)

**Keywords:** Alzheimer's disease, amyloids, solid-state NMR spectroscopy, structure elucidation

## Abstract

Despite its central importance for understanding the molecular basis of Alzheimer's disease (AD), high-resolution structural information on amyloid β-peptide (Aβ) fibrils, which are intimately linked with AD, is scarce. We report an atomic-resolution fibril structure of the Aβ1-40 peptide with the Osaka mutation (E22Δ), associated with early-onset AD. The structure, which differs substantially from all previously proposed models, is based on a large number of unambiguous intra- and intermolecular solid-state NMR distance restraints.

Alzheimer's disease is a progressive neurodegenerative disorder accompanied by fibrillar deposits of amyloid β-peptide in extracellular proteinaceous plaques in the brain.[1] According to the amyloid cascade hypothesis,[2] the formation of Aβ oligomers and fibrils is suspected to be the key event in the development of AD, and the high-resolution structure of Aβ aggregates is crucial for unraveling the molecular basis of the disease. Solid-state NMR spectroscopy has proven potential for the investigation of protein fibrils.[3–5] Several structural models for Aβ fibrils have been proposed based on solid-state NMR restraints,[6–15] including a recent detailed atomistic model of fibrils seeded by a homogenate from diseased brain, representing a β-hairpin in a trimeric arrangement.[14] These models share a common cross-β architecture mostly with in-register parallel pleated sheets (an important exception being the Iowa mutant D23N)[16] and provide strong evidence for polymorphism, namely the propensity of Aβ fibrils to adopt a number of different architectures that may correspond to different disease phenotypes.[14] The self-replication of distinct fibrillar quaternary structures has also been postulated for Parkinson's disease[17,18] and is the structural principle underlying prion strains.[19]

Here we combine solid-state NMR spectroscopy with electron microscopy to obtain the atomic-resolution structure of amyloid fibrils of the familial AD variant Aβ1-40 with the Osaka deletion mutation E22Δ, which is related to early-onset AD.[20] Aβ1-40 E22Δ has been found to be more neurotoxic in rat primary neuron cultures than wild-type Aβ1-40. Also, its aggregates more readily form fibrillar bundles in vitro, with, for the form described here, an unusually high thioflavin T binding capacity.[21,22] Combining mass-per-length data from scanning transmission electron microscopy (STEM) and unambiguous (i.e. the two frequencies of the NMR crosspeak are both assignable to a unique atom) NMR restraints, 26 intra- and 22 intermolecular, we can determine the overall fold of the peptide. In a second calculation based on this data, complemented by 631 automatically assigned medium-, long-range, and intermolecular NMR distance restraints per monomer, which are supported by 2356 peaks in five different NMR spectra, we obtain an atomic-resolution structure of Aβ1-40 E22Δ fibrils.

We first show that the fibril is built by two symmetry-equivalent rigid molecules per layer. Electron microscopy images show that isolated fibril segments can be obtained at an early stage of in vitro fibrillization (Figure 1 a,b). The experimental mass-per-length (MPL) distribution from STEM, shown in Figure 1 c, reveals that the elementary fibril consists of two 4.2 kDa monomers for each layer of β-sheet (ca. 4.85 Å), corresponding to two symmetry-equivalent molecules per layer. NMR sequential resonance assignments are described elsewhere.[23] The spectra used here were recorded on virtually pure samples of a single polymorph. NCA and NCO correlation spectra (Figure 1 d,e) and the DARR correlation spectrum (Figure S1) contain a single set of resonances, demonstrating that all monomers are symmetry-equivalent. All 39 residues are detected in the spectrum, indicating the absence of highly dynamic segments.

**Figure 1:**
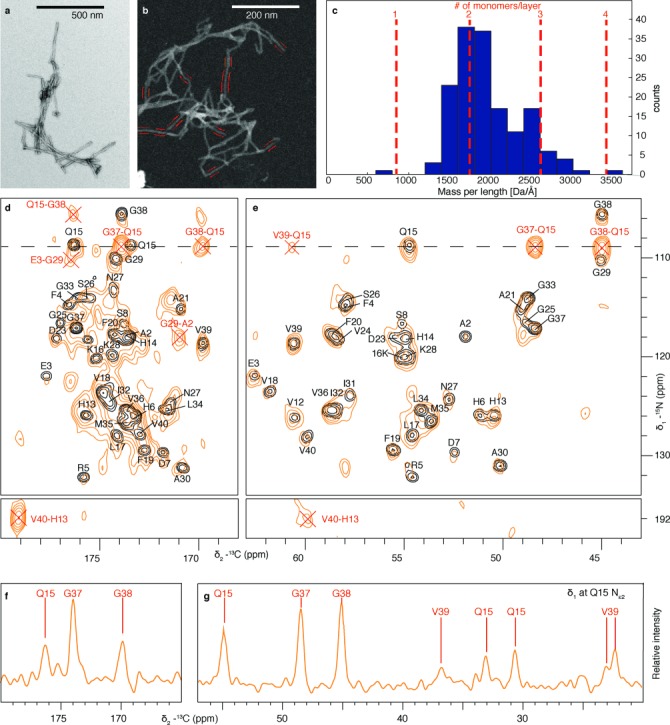
NMR and STEM data establish the basic amyloid architecture. a) Negative-stain TEM image of Aβ1-40 E22Δ fibrils. b) STEM image of unstained, freeze-dried fibrils. Regions that used MPL measurements are marked in red. c) MPL measurement. d,e) The comparison of NCO (d) and NCA (e) NMR spectra of uniformly [^15^N,^13^C]-labeled fibrils (black contours) with mixed [^15^N,^13^C]-labeled fibrils (orange contours) establishes an in-register parallel β-sheet architecture. f,g) Traces taken at the Gln15 Nε2-resonance.

The structure calculation procedure follows established NMR strategies. Distance restraints were extracted from two-dimensional CHHC, PAR, PAIN, DARR, and PDSD spectra[24–27] (Figures 1 d,e and 2 a,b and in Figures S2 and 3). A relatively small number of spectrally unambiguous crosspeaks can provide long-range restraints that already define the structure reasonably well,[3] while the complete information from all ambiguous and unambiguous crosspeaks can be used to obtain a more precise structure.[4] For amyloids, structurally meaningful intermolecular distances are abundant and can be similar to intramolecular distances.[4] It is therefore necessary to establish the inter- or intramolecular nature of NMR restraints using samples with specific isotope-labeling patterns.

**Figure 2:**
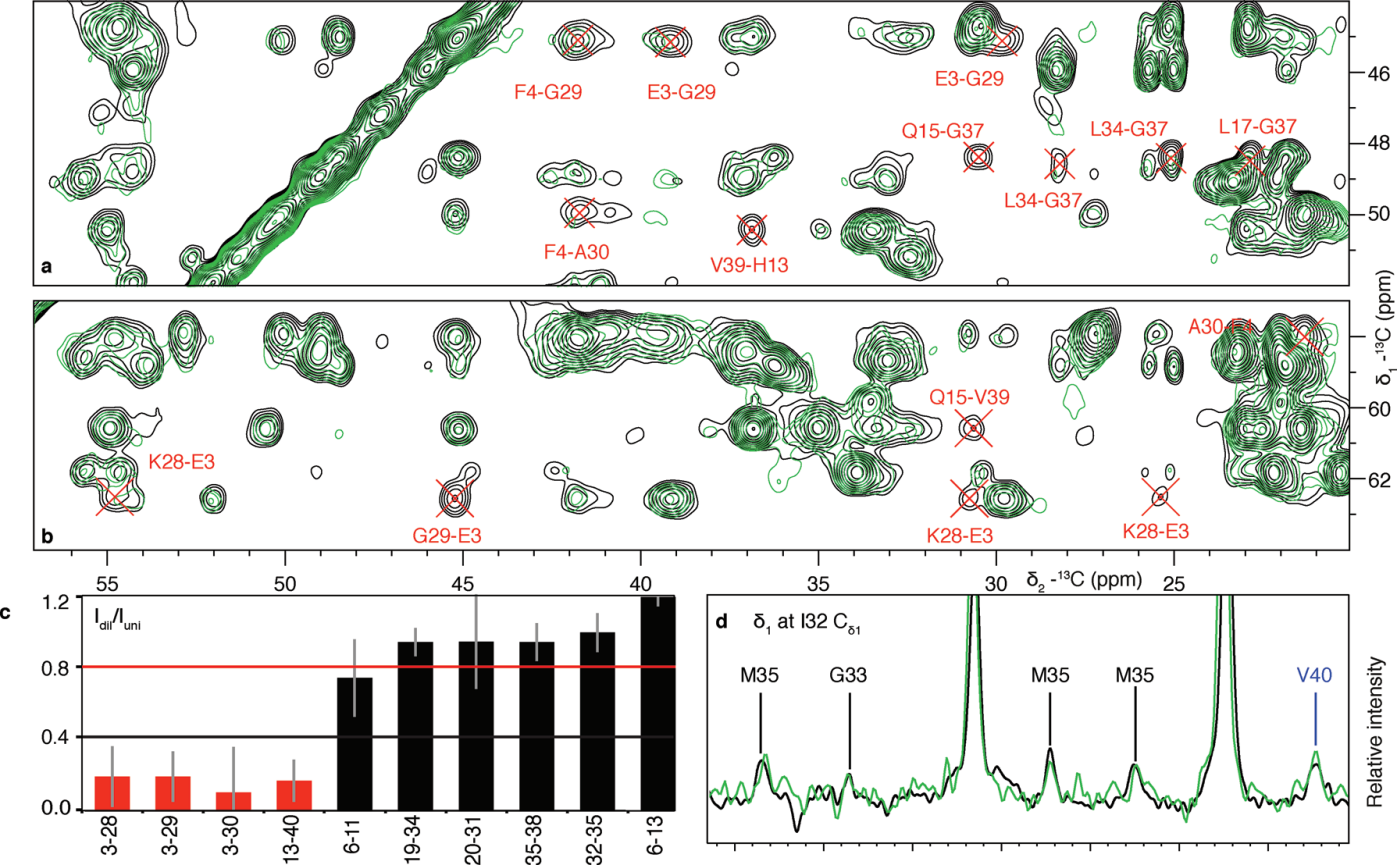
Extracts of NMR spectra that distinguish intra- from intermolecular contacts. a,b) Overlay of PAR spectra recorded on a uniformly [^13^C]-labeled sample (black contours) and on a sample of 20 % uniformly [^15^N,^13^C]-labeled protein and 80 % unlabeled material (green contours). Details are given in Figure S5. c) Intensity ratios of crosspeaks in dilutely and uniformly labeled samples. Inter- and intramolecular correlations, expected to be attenuated to 20 % and not attenuated, are shown as red and black bars, respectively. The full statistics are shown in Figure S5. d) Trace extracted at the Ile32 Cδ1-resonance from PAR spectra of uniformly (black) and dilutely labeled (green) samples. All cross peaks of this resonance are intramolecular.

We first addressed the question of the register of β-strands stacking into sheets; the observation of highly similar N–Cα and N–C′ cross signals (Figure 1 d,e) in NCA/NCO spectra recorded on a uniformly labeled sample (where correlations are of intraresidue nature) and PAIN spectra recorded on a 1:1 mixed [^15^N,^13^C]-labeled sample (where they exclusively arise from intermolecular interactions between adjacent layers of β-sheet) revealed a parallel in-register stacking of the β-sheets.

Then we looked for further peaks that are clearly intermolecular. PAIN spectra of the mixed sample feature only intermolecular ^15^N–^13^C correlations and, besides signals connecting adjacent layers in the cross-β arrangement, we found strong signals not explained by register contacts (e.g. red crosses in Figure 1 d,e). Five of these additional peaks provide spectrally unambiguous intermolecular restraints (Table S1).

Further information about intra- and intermolecular packing in the fibril was obtained by comparing the crosspeak intensity of uniformly and dilutely labeled samples (labeled monomers diluted in unlabeled ones) in CHHC, PAR, and DARR spectra (Figure 2 a,b,d and Figure S4). Intermolecular peaks are attenuated by roughly the dilution ratio (here 1:4), while intramolecular peaks remain constant. A detailed comparison of the diluted and nondiluted samples is given in Figure S4 and the resulting attenuation factors are given in Figure 2 c and Figure S5. This analysis yielded restraints that are spectrally unambiguous and unambiguously either intermolecular or intramolecular. A total of 48 restraints were extracted from the spectra and classified as 22 intermolecular and 26 intramolecular restraints (Table S1 and Figure S6).

An initial “manual” structure calculation with CYANA[28] used only the manually identified 48 unambiguous restraints (Table S1) in addition to hydrogen bonds defining the β-sheets (Figure 3 b) and 56 dihedral angle restraints from TALOS+.[29] The resulting backbone structure is shown in Figure 3 a; for details see the Supporting Information. Intermolecular restraints (red in Figure 3) can represent lateral contacts between two molecules if all β-sheets form a planar arrangement with the β strands running perpendicular to the fibril axis, or stagger contacts if one monomer extends across two planes.[8,30] Manual structure calculations resulted exclusively in all-lateral solutions. Forcing staggering led to higher target functions (Figure S8). The C-terminal hydrophobic residues (Ile32–Val40) do not form a straight β-sheet but fold back such that they fulfill the unambiguously intramolecular contacts Ile32–Met35 (Figure 2 d and Figure S5C) and Met35–Gly38 (Table S1 and Figure S4E). Further support for this feature, which is specific to the observed fold, is provided by the following observations not used in the calculation to independently cross-validate: 1) The fold predicts an intramolecular Ile32–Val40 contact (dashed line in Figure 3 a) whose existence is confirmed by the PAR spectrum in Figure 2 d: the signal marked “V40” and assigned to Ile32δ1–Val40γ1/2 is clearly visible and *not* attenuated by dilution. The alternative assignment possibilities for this peak led to restraint violations. 2) The fold explains all 21 spectrally unambiguous peaks identified in the [2-^13^C]-glucose PDSD spectrum listed in Table S1 (see the Supporting Information).

**Figure 3:**
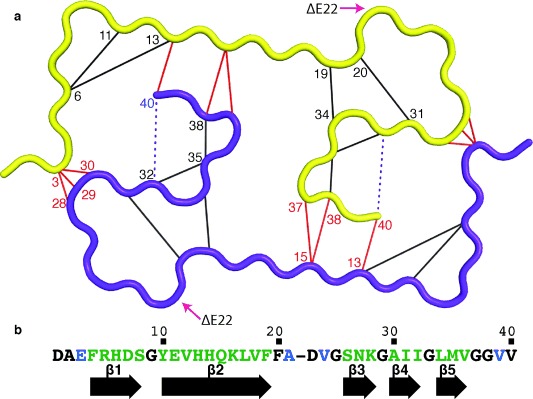
a) Backbone structure of Aβ1-40 E22Δ calculated on the basis of unambiguous distance restraints (solid red and black lines). Blue dashed contacts are discussed in the text but were not used in the calculation. b) Register hydrogen bonds between β-strands entered into the calculation (green lettering). Blue: additional residues with TALOS+ predictions used in the structure calculation.

Automatic structure calculation was conducted using, in addition to the information from the manual calculation, automatically picked peak lists from the CHHC, PAR, PDSD, and two PAIN spectra (Table S2) for iterative assignment by CYANA (Table S2). This procedure yielded the structure shown in Figure 4. The quaternary structure of the fibril is defined by two interlaced protofilaments and resembles a cinnamon roll. Figure 4 b displays the NMR bundle representing the 20 lowest-energy structures. The structure is deposited in the PDB database with ID 2mvx, the chemical shifts in the BMRB with ID 25289. The cross section of the fibril can be approximated by a rectangle that is closed by intermolecular salt bridges between the Glu3 and Lys28 side chains. This salt bridge seems not to be present in wild-type Aβ fibril models, where Lys28 was often found to interact with Asp23.[11,30] Figure 4 c shows the positioning of the side chains in the fibril and reveals that the very center is filled with exclusively hydrophobic residues, indicating that hydrophobic interactions strongly contribute to fibril stability. Notably, four charged residues, including the C-terminal carboxylate of Val40, are located inside the fibril core, and form a network of salt-bridge interactions (Figure S7). This network of salt bridges energetically stabilizes the turn region from Gly9 to His13, as well as the quaternary arrangement of monomers. The evidence against threefold symmetry is discussed in Figure S9.

**Figure 4:**
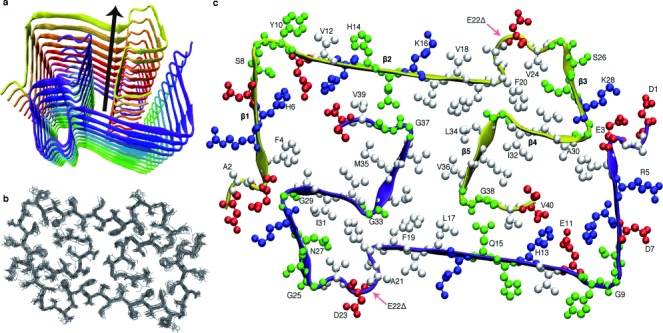
Representation of the 3D structure of Aβ1-40 E22Δ calculated from NMR-derived distance and torsion-angle restraints. The structure is deposited in the Protein Data Base with accession number 2mvx. a) Schematic view of the lowest-energy conformer of a Aβ1-40 E22Δ bi-decamer (2×10 monomers). The symmetry axis (arrow) coincides with the long axis of the fibril. b) NMR bundle of the middle layer only. c) Cross section of the fibril hydrophobic residues are colored white, negatively charged residues red, positively charged residues blue, and polar ones (and Gly) green.

In summary, we present the atomic-resolution structure of amyloid β fibrils formed by the Osaka deletion mutant, which is linked to early-onset AD. The structure is highly ordered and its stability can be rationalized by building principles known from other classes of proteins. The deletion mutation, which is located in vicinity of several other familial mutations (Arctic, E22G; Dutch, E22Q; Flemish, A21G; Italian, E22K; Iowa, D23N), is found in a turn in the structure. The overall fold is thus, in principle, also accessible to the wild-type Aβ1-40 peptide and those mutants. In fact, it has recently been shown that Aβ1-40 E22Δ fibrils can impose their structure, in a prion-like fashion, on wild-type Aβ1-40.[31] Our work thus provides a structural basis for further work towards understanding Aβ fibril formation, propagation, and drug-binding studies[32] on a molecular level.
