# Supplementary material for: Atomic-Resolution Three-Dimensional Structure of Amyloid β Fibrils Bearing the Osaka Mutation
Source: Angew Chem Int Ed Engl. 2014 Nov 13;54(1):331–5. doi: 10.1002/anie.201408598 (PMC4502972; doi:10.1002/anie.201408598)
Supplement: Supplementary file 1 [file anie0054-0331-sd1.pdf]

Angewandte Chemie
Eine Zeitschrift der Gesellschaft Deutscher Chemiker

Supporting Information
© Wiley-VCH 2014
69451 Weinheim, Germany

Atomic-Resolution Three-Dimensional Structure of Amyloid β Fibrils Bearing the Osaka Mutation**

Anne K. Schütz, Toni Vagt, Matthias Huber, Oxana Y. Ovchinnikova, Riccardo Cadalbert, Joseph Wall, Peter Güntert, Anja Böckmann, Rudi Glockshuber,* and Beat H. Meier**

anie_201408598_sm_miscellaneous_information.pdf

Contains: Material and Methods

Figures S1-S7

Tables S1-S3

The atomic coordinates and chemical-shift assignments have been deposited in the PDB with accession number (to be filled in).

Materials and Methods

Protein expression and purification

Plasmid construction for recombinant expression of Aβ1-40 E22Δ in *E. coli* as well as protein expression, purification and fibril production have been described in detail[1,2]. The protein monomer concentration used here for fibrillization was 60 µM, in a reaction buffer of 10 mM NaH_2PO_4-NaOH pH 7.4, 100 mM NaCl, at 37 °C, 700 rpm magnetic stirring, 80 min incubation time.

Mass per length measurements using STEM

An aliquot of the fibrillization mixture was taken after 90 min and immediately washed using ammonium acetate buffer, blotted onto custom-made EM grids and freeze-dried. The remaining fibrils were washed and ultracentrifuged. The NMR spectrum of this sample was identical to the one shown in Fig. S1, demonstrating that the fibrils used in STEM measurements are identical to fibrils used for NMR spectroscopy.

STEM measurements were carried out at Brookhaven National lab. STEM data were analyzed using PCMass32 (http://www.bnl.gov/biology/stem/). The criteria for manual selection of filaments were that filaments were straight and regular in appearance, not fragmented, well-isolated, with a clean background and not embedded in salt, monomer or denatured protein. 159 manual mass measurements were conducted and a histogram is shown in Fig. 1c. Even if a a value of two monomers per layer two is clearly predominant, we do not yet completely exclude three on the sole basis of the STEM data (see Fig. S9 for NMR evidence supporting the value of two).

NMR structure determination

Sample preparation

Fibril samples were centrifuged for 45 min at 30,000 g and 4 °C, washed with deionized water and centrifuged again. The pellet containing approximately 10-15 mg Aβ1-40 E22Δ fibrils was resuspended in 800 µL deionized

water and packed into a 3.2 mm ZrO_2 rotor (Bruker Biospin) using ultracentrifugation and a home-made filling device[3], at 150000 g for 16 hours.

Data recording

All NMR spectra were recorded on a Bruker Avance II+ at a static magnetic field of 20.0 T using a Bruker 3.2 mm triple-resonance probe spinning at 19 kHz (except for the PDSD spectrum of the [2-^{13}C glucose]-labeled sample, which was recorded at 11.5 kHz MAS). Sample temperatures were 5-15° C as determined from the water resonance position[3]. The NMR experiments recorded on various samples and the corresponding experimental parameters are listed in Table S3. The spectra of the uniformly [^{15}N,^{13}C]-labeled sample were referenced to DSS and the shifts of all other samples were referenced to this sample. All spectra were processed in TopSpin 2.1 (Bruker Biospin). Spectra were zero-filled to the next power of two in data points. Spectra used for dilution analysis were zero-filled, before Fourier transform, to 16k x 16k data points.

Analysis of the spectra

Spectra were analyzed and annotated using CcpNmr version 2.2.2[4]. To account for small chemical shift variations between fibril batches, separate shift lists were maintained for every sample. For CHHC assignment, ^{13}C shifts were translated to ^{1}H assignments of the closest proton (in terms of the number of bonds separating ^{13}C and ^{1}H).

Hydrogen-bond restraints were identified when three consecutive negative secondary chemical shifts were observed[5] with the exception of glycine residues, for which no β-strands were imposed. Their position is indicated in Fig. 3C. Dihedral angle restraints were predicted from the ^{13}C and ^{15}N chemical shifts using the TALOS+ software[6] and only predictions rated as “good” were used. Almost all glycines produced TALOS+ warnings and all Gly residues were excluded from the torsion-angle restraints.

For manual restraint identification, isolated correlations that could be spectrally unambiguously assigned to single resonances in both dimensions within a tolerance of 0.2 ppm were identified in the following spectra (and samples): DARR 400 ms, PAR 8 ms, CHHC 500 us (U-[^{13}C]-labeled and dilutely labeled), PAIN 6 ms (U-[^{15}N,^{13}C]- and mixed [^{15}N]:[^{13}C]-labeled) and PDSD 4 s ([^{15}N, 2-^{13}C glucose]-labeled). PAIN spectra recorded on the mixed sample show exclusively intermolecular peaks. Signal-to-noise ratio permitting, the remaining restraints were subjected to further analysis in order to identify their inter- or intramolecular nature. To this purpose, the spectrally unambiguous peaks identified in DARR 400 ms, PAR 8 ms, CHHC 500 µs (U-[^{13}C]) were compared to the equivalent spectra recorded on the dilutely labeled sample. Representative traces are shown in Figure S4. Intensities were normalized for each residue using unambiguous intramolecular peaks and the positions of the two traces were adjusted such that they were at the respective peak maximum. The intensity ratios of peak maxima I_{dil}/I_{uni} were evaluated and the results are displayed in Figure S5, and per residue averages are summarized in Fig. 2C. Errors were estimated from the noise level and represent standard deviations. Peaks were subsequently classified as intramolecular if the intensity ratio was $I_{dil}/I_{uni} \geq 0.8$ with a lower error margin above 0.4. Conversely, peaks were classified as intermolecular if $I_{dil}/I_{uni} \leq 0.4$ with an upper error margin below 0.8. In all other cases no classification was done. The corresponding restraints were translated to upper distance restraints with values as listed in Table S2. This data analysis yields a list of unambiguous restraints, spectrally as well as with respect to their classification as intra-/intermolecular, as listed in Table S1.

Optimal upper distance limits for each experiment were determined following the L-curve approach[7]. The optimum distances found are given in Table S2 and they are very similar to the ones in[7].

Peak lists for automatic structure calculation were generated using automatic peak picking performed in CcpNmr. Spinning-sideband positions were removed manually.

Structure calculation

Structure calculations follow established principles, namely identifying a large number of interatomic distances and converting them into upper distance restraints for triangulation were performed in CYANA 3.96. All manual and automatic restraints were given the same relative weight (1.0) except for hydrogen-bond upper and lower distance limits (relative weight 10.0). Hydrogen bonds were implemented as lower and upper distance limits from H^N and N to O of 1.8-2.0 Å and 2.7-3.0 Å, respectively. Fibril structure calculation was performed for a penta-dimer (5x2 monomers), tri-dimer (3x2) or deca-dimer (10x2). No significant differences were detected, as expected[7]. For comparison, tri-trimers (3x3) and penta-trimers were calculated. Symmetry was enforced for all molecules by minimizing dihedral angle-differences and differences of symmetry-related intermolecular distances[8]. Restraint assignment in the automatic calculation was allowed within monomers and laterally between monomers and assignments were enforced to be C2(C3)-symmetric with respect the fibril long axis. His6 and His13 were implemented as positively charged as indicated by the sidechain nitrogen chemical shifts. The N-terminal aspartate and the C-terminal valine residue were treated as positively and negatively charged, respectively.

The following restraints were used for **manual structure calculation** (numbers are given per monomer): 31 manual restraints (upl) supported by the 48 unambiguous cross peaks listed in Table S1 with 15 intermolecular upl's entered as either staggered or lateral, 28 pairs of ϕ/ψ dihedral angle restraints for the residues in green or blue lettering in Fig 3c; 19 hydrogen bonds between residues in green lettering in Fig. 3C. Upper distance restraints of 5.5, 6.0, 5.0 and 7.0 Å were applied for restraints from CHHC, PAR, PAIN and DARR spectra. 500 structures were annealed in 35,000 torsion-angle dynamics steps. The structure with the lowest target function had no violations and is shown in Fig. 3. All 16 permutations of the possible relative directions of the five β-sheets were evaluated in separate CYANA structure calculations. Only a single arrangement of β-sheets yielded a violation-free solution. This solution represents a planar arrangement with all intermolecular restraints fulfilled within the plane of Fig. 3. Attempts to use staggered arrangements to fulfill the restraints by alternative motifs always lead to violations. (see Figure S8). This observation is in line with the existence of a significant number of intramolecular restraints along the entire sequence (black lines in Fig.3), which are impossible to fulfill if the molecule is not contained within a single plane.

As a cross check additional 21 spectrally unambiguous restraints from the 4 s PDSD spectrum where added to the manual calculation (for a list see Table S1). These restraints were not further used in other calculations. Using a distance limit of 8.5 Å CYANA returns the fold of Fig. 3 without restraint violation. The distance limit of 8.5 Å corresponds to the expectations for this very long mixing time (4 s) and exceeds the limits used for the other spectra (Table S2).

For **automatic** structure calculation the same input as for manual structure calculation was used. In addition, restraints from five spectra were automatically assigned by CYANA: peak lists of the PAR 8 ms, CHHC 500 μs, and

PDSD 4 s, two PAIN 6 ms spectra (with the ^{13}C carrier on aliphatic and on aromatic resonances during the PAIN transfer) using chemical-shift lists of the respective samples. For the final calculations, additional restraints for salt bridges were generated for oppositely charged residues for which ^{13}C-^{13}C or ^{13}C-^{15}N side chain contacts were observed in the spectra. Upper limit restraints were introduced for Glu3 Oε1/2 to Lys27 Nζ (5 Å); His6 Nε2 to Glu11Oε1/2 to (4 Å); Glu11Oε1/2 to His13 Nε2 (4 Å); His13 Nδ1 (4 Å) to Val40 O1/2 (Fig. S7). 500 structures were annealed in 35,000 steps in seven assignment cycles. All 16 relative orientations of the 5 β-sheets were explored, as in the manual calculation, and again the same arrangement had clearly the lowest target function. For the final calculation, the intermolecular restraints were defined to be intra-lateral (excluding inter-lateral register peaks). The structure obtained is however identical, within our precision, to the one obtained when these contacts were allowed to be either intra- or interlateral.

To obtain an NMR bundle that realistically depicts the precision of the structure determination we used an approach that also considers the random seed-dependence of the CYANA calculation with automatic distance restraint assignment by constructing a bundle of the best structures from 20 different bundles each calculated from a different "random" initial condition determined by the initial "seed" value of CYANA. This structure bundle is deposited in the PDB protein databank (http://www.rcsb.org) under the ID (to be inserted). While the backbone rmsd of a single bundle was 0.2 ± 0.1 Å, the bundle of 20 bundles had an rmsd of 0.8 ± 0.3 Å. The heavy atom rmsd were 0.6 ± 0.1 Å and 1.1 ± 0.3 Å, respectively. Variations between the bundles can result from different assignments that have been chosen for highly ambiguous cross peaks in different CYANA runs.

Out of all 20 bundles, the structure with the lowest CYANA target function is shown in Fig. 4. In the best structure out of 500 structures, residues were distributed as follows on the Ramachandran diagram: 82.5% most favored, 14.3% additionally allowed, 3.2% generously allowed, 0% disallowed. For this structure, the PAR spectrum was back calculated. As shown in Fig. S10, the prediction explains the spectrum very well.

Supplementary References

[1] V. H. Finder, I. Vodopivec, R. M. Nitsch, R. Glockshuber, *J Mol Biol* **2010**, *396*, 9–18.

[2] O. Y. Ovchinnikova, V. H. Finder, I. Vodopivec, R. M. Nitsch, R. Glockshuber, *J Mol Biol* **2011**, *408*, 780–791.

[3] A. Böckmann, C. Gardiennet, R. Verel, A. Hunkeler, A. Loquet, G. Pintacuda, L. Emsley, B. H. Meier, A. Lesage, *J Biomol NMR* **2009**, *45*, 319–327.

[4] T. J. Stevens, R. H. Fogh, W. Boucher, V. A. Higman, F. Eisenmenger, B. Bardiaux, B.-J. van Rossum, H. Oschkinat, E. D. Laue, *J Biomol NMR* **2011**, *51*, 437–447.

[5] M. Huber, O. Y. Ovchinnikova, A. K. Schütz, R. Glockshuber, B. H. Meier, A. Böckmann, *Biomol NMR Assign* **2014**, ahead of print.

[6] Y. Shen, F. Delaglio, G. Cornilescu, A. Bax, *J Biomol NMR* **2009**, *44*, 213–223.

[7] H. van Melckebeke, C. Wasmer, A. Lange, E. Ab, A. Loquet, A. Böckmann, B. H. Meier, *J Am Chem Soc* **2010**, *132*, 13765–13775.

[8] Y. J. Lin, P. Güntert, D. K. Kirchner, *J Magn Reson* **2012**, *222*, 96–104.

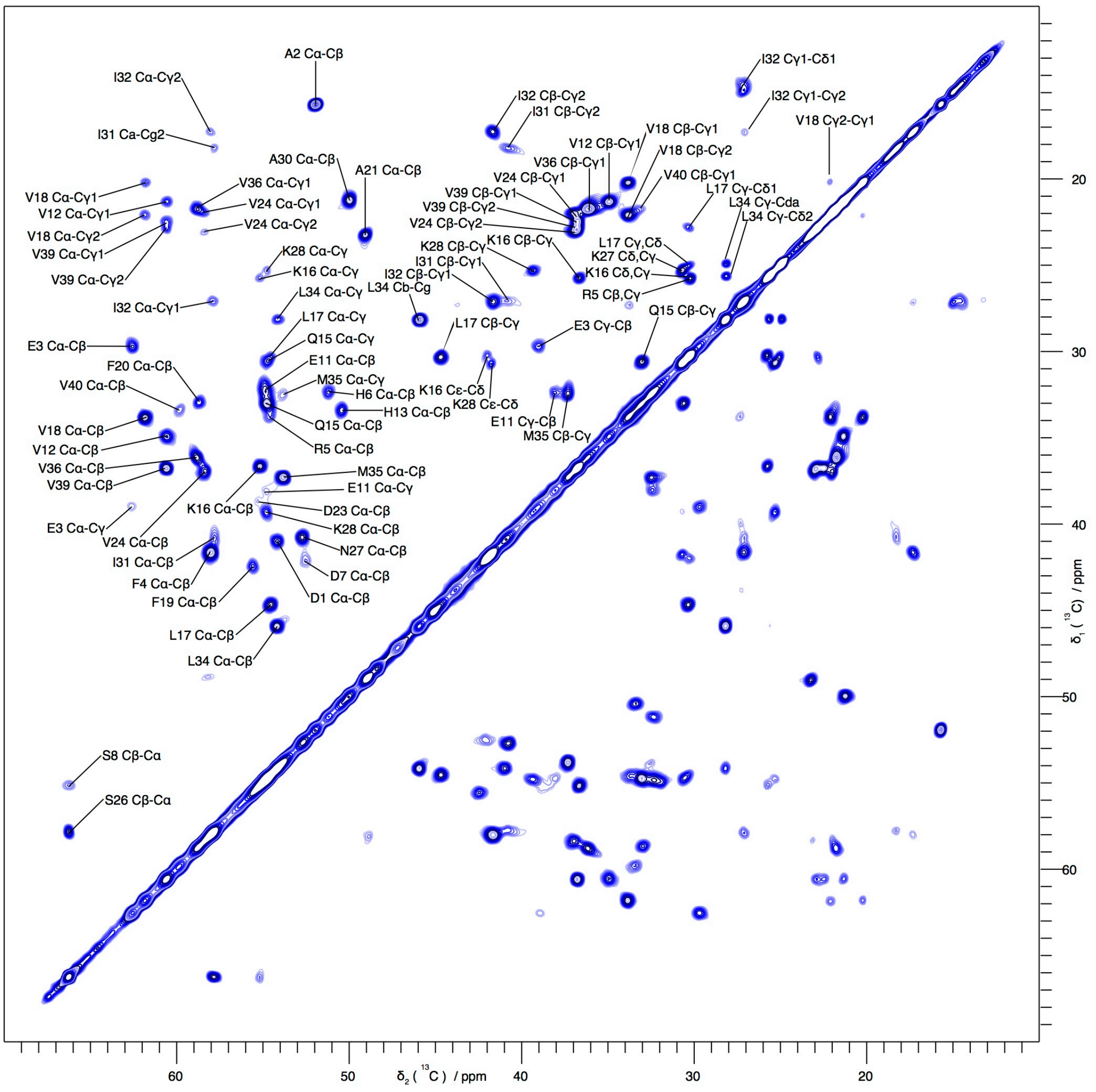

Fig. S1.

Assignment of ^{13}C intra-residue correlations. Two-dimensional ^{13}C-^{13}C correlation spectrum (DARR 15 ms) recorded on uniformly [^{13}C]-labeled fibrils at 20.0 T (850 MHz proton frequency) at 19 kHz MAS. The measurement time was 3 h. At short mixing times, mostly one-bond correlations are detected. The sequential assignments are provided. A single set of resonances is observed for all 39 residues and no polymorphism could be detected in this sample. The ^{13}C,^{13}C restraint spectra (DARR 400ms, PAR 8ms, CHHC 500 us) were recorded on this fibril sample.

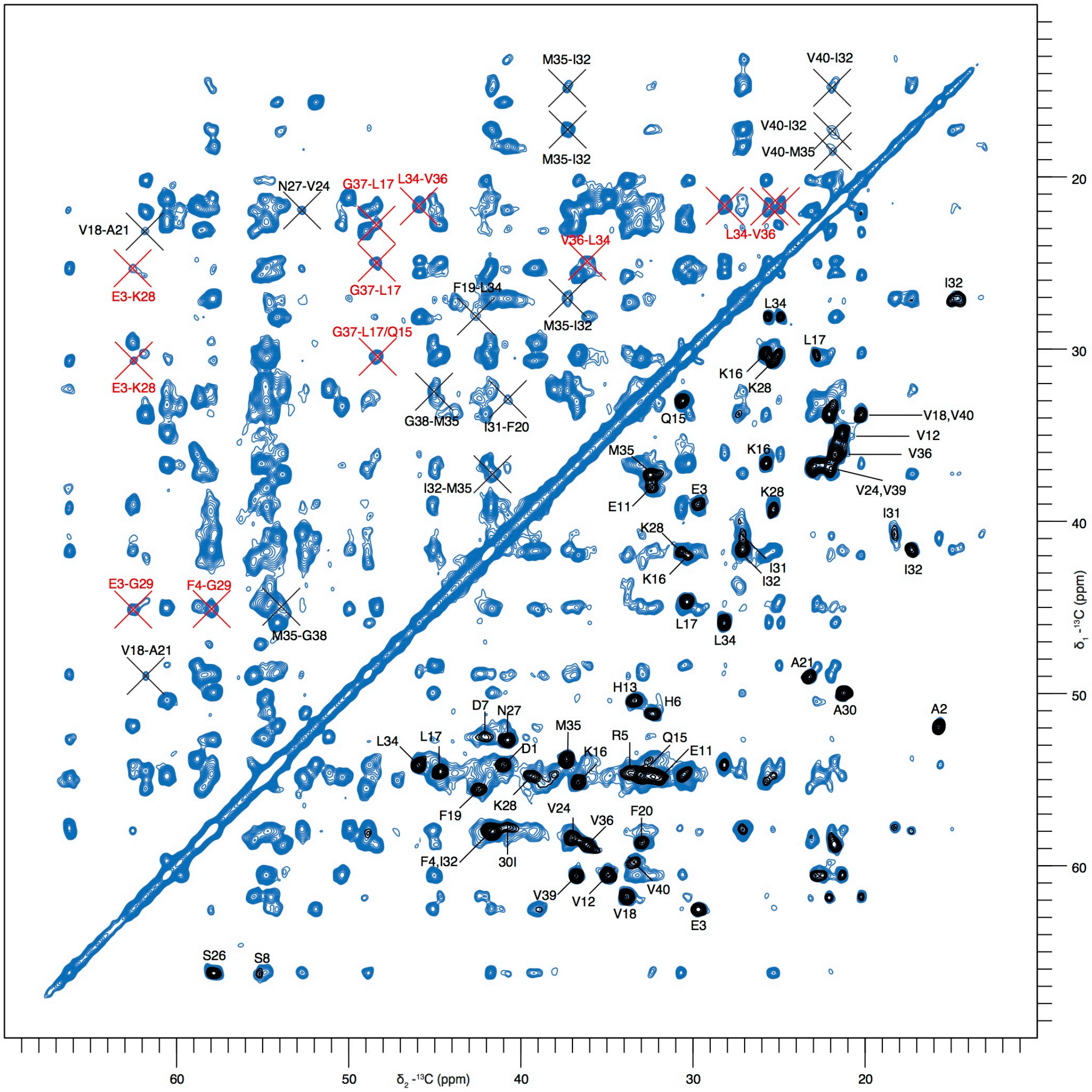

Fig. S2

CHHC restraint spectrum. Blue contours: aliphatic region of a CHHC spectrum with 500 μs mixing time recorded on uniformly [^{13}C] labeled fibrils at 19 kHz MAS at a static magnetic field of 20.0 T (850 MHz proton frequency). The measurement time for this spectrum was 34 hours. The DARR 15 ms spectrum (from Fig. S1) is shown in black contours below the diagonal including assignments of intra-residue correlations of directly bonded atoms. Above the diagonal in the CHHC spectrum, selected medium- and long-range contacts in the CHHC spectrum are labeled (intramolecular in black, intermolecular in red). A total of 7 spectrally unambiguous long-range restraints were manually identified in the CHHC spectrum (Table S1) and an additional 388 medium-range, 101 long-range and 399 inter-molecular restraint assignments were automatically identified by CYANA.

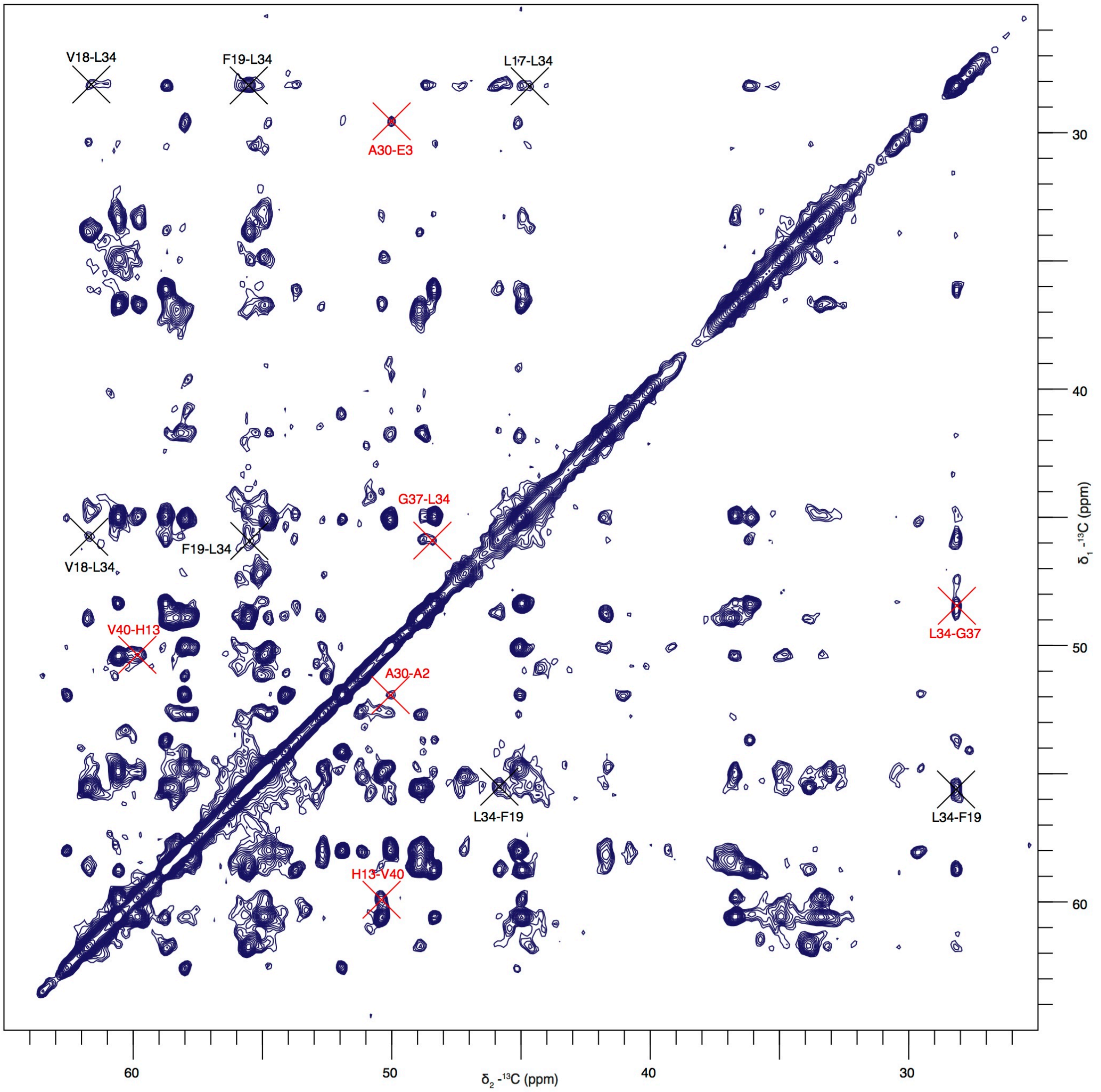

Fig. S3

PDSD spectrum. Aliphatic correlation region of a PDSD spectrum recorded with 4 s mixing time on sparsely labeled [^{15}N, 2-^{13}C-glucose] fibrils at 11.5 kHz MAS at a static magnetic field of 20.0 T (850 MHz proton frequency). The measurement time was 181 h.

Spectrally unambiguous long-range contacts are labeled in black if they are intramolecular, in red if they are intermolecular. A total of 29 spectrally unambiguous long-range restraints were manually identified in this spectrum (Table S1) and an additional 238 medium-, 66 long-range and 284 intermolecular restraint assignments were automatically identified by CYANA.

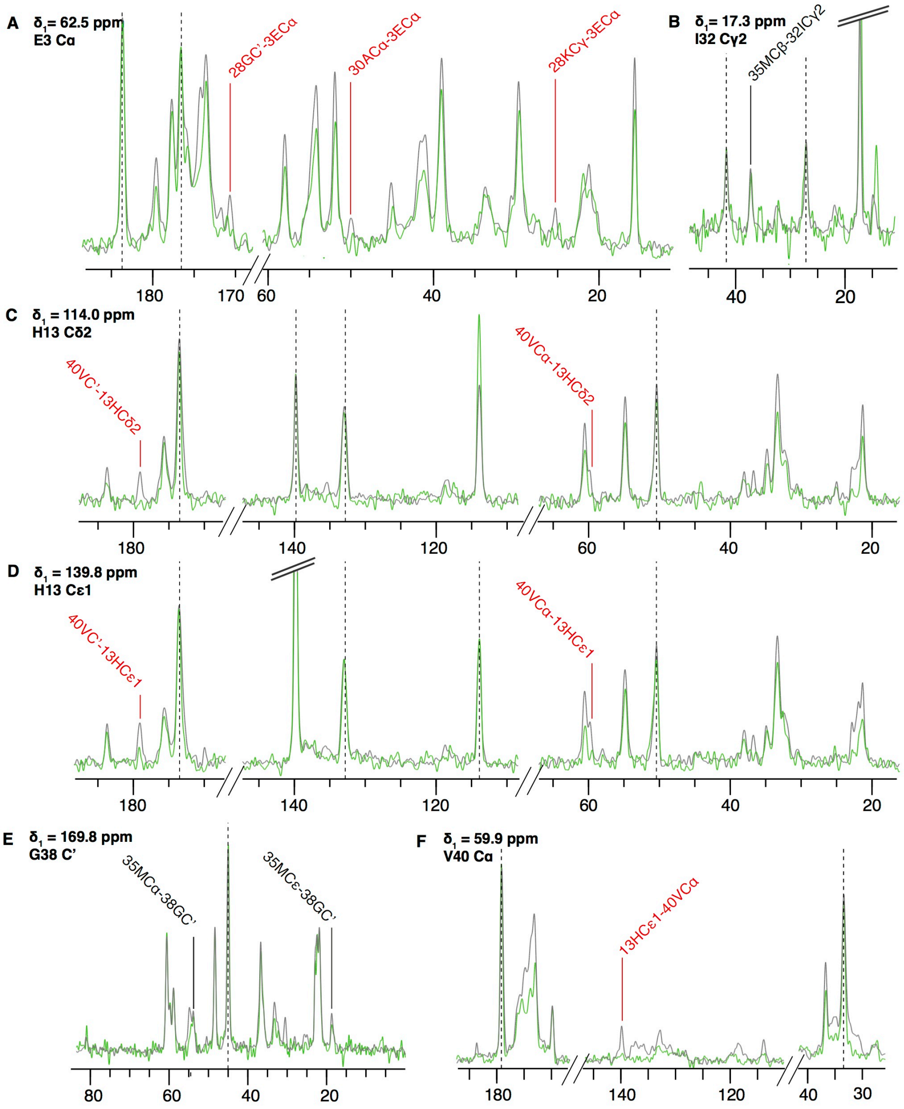

Fig. S4

Traces from 2D spectra recorded on dilutely and uniformly labeled samples. Traces were taken at the positions of spectrally unambiguous resonance frequencies in δ1 from DARR (A, C-F) and CHHC (B) spectra recorded on dilutely (green) and uniformly labeled (black) labeled fibrils. DARR and CHHC spectra were acquired at 19 kHz MAS at 20.0 T (850 MHz proton frequency). The measurement times for DARR/CHHC were 25/34 h for the uniformly and 211/205 h hours for the dilutely labeled sample. All labeled resonances are unambiguous. Intermolecular contacts are attenuated upon dilution and are labeled in red. Intramolecular contacts remain unchanged upon dilution and are labeled in black. Calibration was done using the signals indicated by broken lines, which correspond to intraresidue correlations.

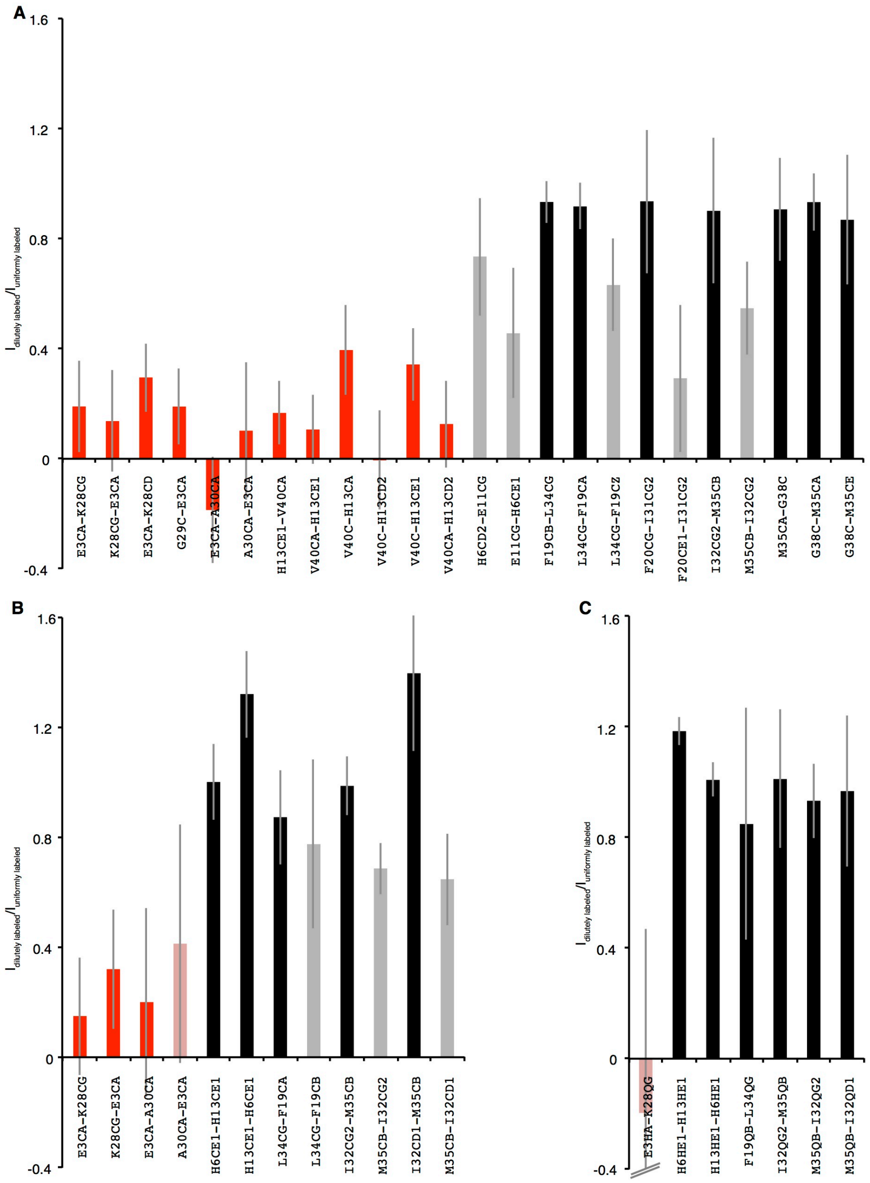

Fig. S5

Dilution analysis of unambiguous restraints. Intensity ratio of unambiguous restraints in dilute versus uniformly labeled fibrils identified in DARR (A), PAR (B) and CHHC (C) restraint spectra. Contacts classified as inter- or intramolecular are depicted in red and black respectively. They are shown in light colors if no classification was performed. The color code was then deduced from other restraints that represent the same residue pair. Peaks were classified as intramolecular if the intensity ratio was $I_{dil}/I_{uni} \geq 0.8$ with a lower error margin above 0.4. Conversely, peaks were classified as intermolecular if $I_{dil}/I_{uni} \leq 0.4$ with an upper error margin below 0.8. In other cases no classification was done.

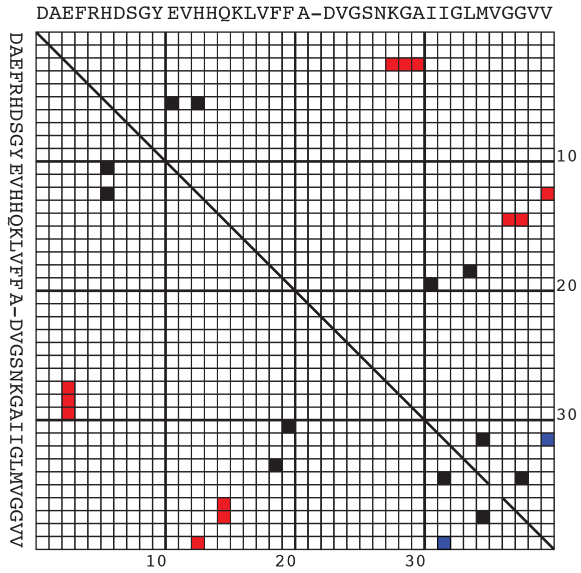

Fig. S6
Interaction matrix with spectrally unambiguous inter- and intramolecular contacts indicated in red and black, respectively. The blue contact does not lead to spectrally unambiguous cross-peaks and is therefore not used for the manual structure calculation, but allows to cross-check the structure.

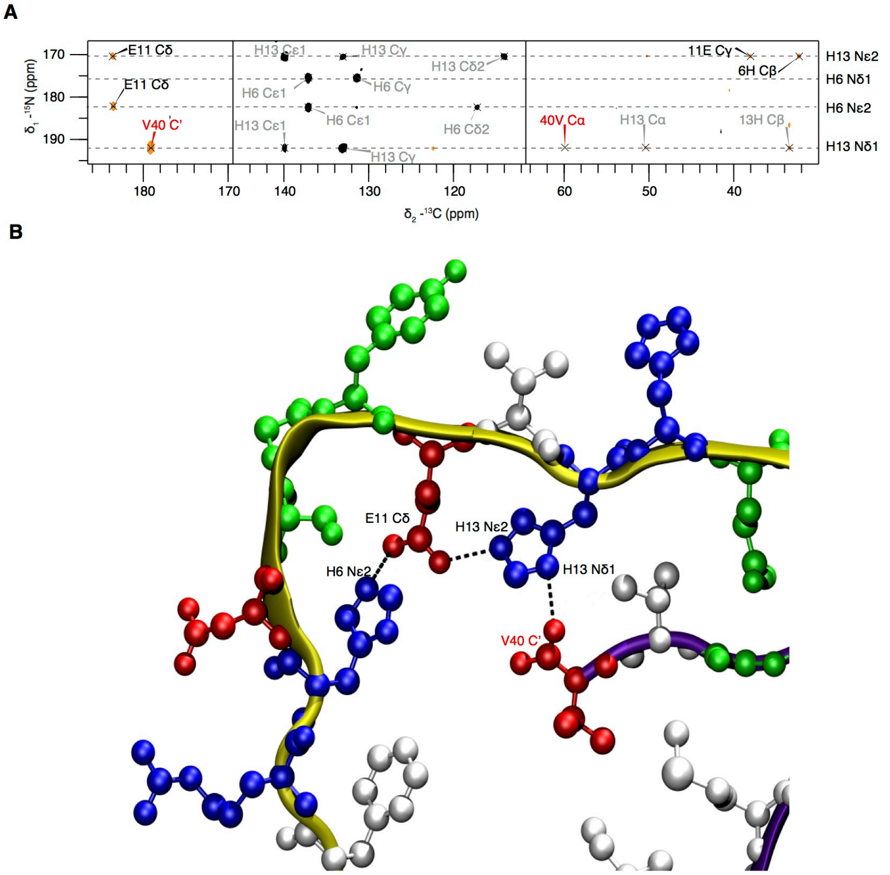

Fig. S7

Saltbridge network in the fibril core. (**A**) Extracts of 2D NCA (black contours, uniformly [^{15}N,^{13}C]-labeled sample) and PAIN (orange contours, mixed [^{15}N]:[^{13}C]-labeled sample) spectra in the region of histidine side chain resonance frequencies, showing cross-peaks involving His6, Glu11, His13 and Val40 correlations. Intra-residue correlations in the NCA spectrum are labeled in grey, lateral contacts between monomers in the PAIN spectrum are shown in red and intra-monomer contacts in black (which represent register contacts in the mixed sample). (**B**) The three ionic interactions revealed by these cross-peaks are indicated on the atomic structure. They are located in a turn-region at one of the corners of the square that defines the fibril cross-section.

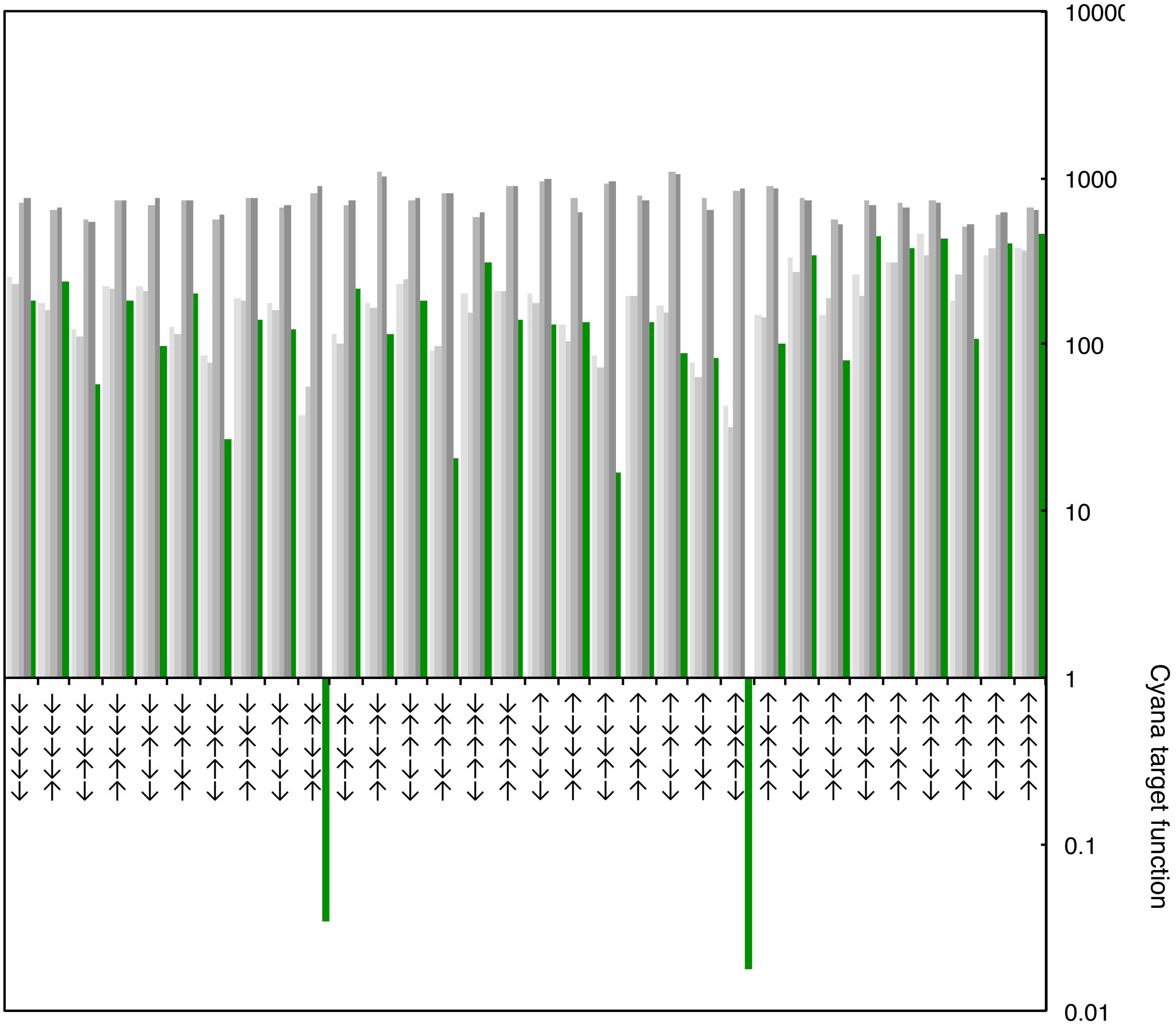

Fig. S8

The diagram shows the CYANA target functions (TF) for manual calculation runs with $2^5=32$ possible relative directions of the five β-sheets (see Figure 3 for definition) in the fibrils.

For each β-sheet configuration, the four grey bars indicate the TF of fibril folds with one group of intermolecular contacts assumed as lateral and the other as staggered: 3-28/29/30 contact lateral, 13/15-37/38/40 staggered +1; 3-28/29/30 contact lateral, 13/15-37/38/40 staggered -1; 3-28/29/30 staggered +1, 13/15-37/38/40 lateral; 3-28/29/30 staggered -1, 13/15-37/38/40 lateral.

The green bars indicate calculations in which all intermolecular contacts are assumed to be lateral. Only for these calculations does CYANA find two structures with no violations and a target function close to zero. These two solutions are symmetry-equivalent.

Only all-lateral definition of the two molecules per layer, as shown in Fig.3 and 4 are supported by the unambiguous restraints.

A

```
 7 ASP  CA      31 ILE  CG2
27 ASN  CA      31 ILE  CG2

32 ILE  CD1     35 MET  CB

32 ILE  CG2     35 MET  CB

13 HIS  CA      17 LEU  CD1
13 HIS  CA      39 VAL  CG1

 6 HIS  CB      32 ILE  CG2
11 GLU  CB      32 ILE  CG2
35 MET  CG      32 ILE  CG2

 6 HIS  CB      32 ILE  CD1
11 GLU  CB      32 ILE  CD1
35 MET  CG      32 ILE  CD1

18 VAL  CG1     31 ILE  CG2
24 VAL  CG2     31 ILE  CG2
40 CVAL CG1     31 ILE  CG2

18 VAL  CG1     32 ILE  CG2
24 VAL  CG2     32 ILE  CG2
40 CVAL CG1     32 ILE  CG2

18 VAL  CG1     32 ILE  CD1
24 VAL  CG2     32 ILE  CD1
40 CVAL CG1     32 ILE  CD1

18 VAL  CG2     21 ALA  CB

17 LEU  CD1     34 LEU  CG
39 VAL  CG1     34 LEU  CG

18 VAL  CG1     32 ILE  CD1
23 VAL  CG1     32 ILE  CD1
40 CVAL CG1     32 ILE  CD1

32 ILE  CG2     36 VAL  CG2
32 ILE  CG2     40 CVAL CG1

 6 HIS  CB      32 ILE  CG2
11 GLU  CB      32 ILE  CG2
35 MET  CG      32 ILE  CG2
```

```
15 GLN  CG      34 LEU  CG
16 LYS  CD      34 LEU  CG
17 LEU  CG      34 LEU  CG

31 ILE  CG1     35 MET  CB
32 ILE  CG1     35 MET  CB

11 GLU  CB      31 ILE  CG1
11 GLU  CB      32 ILE  CG1
35 MET  CG      31 ILE  CG1
35 MET  CG      32 ILE  CG1

 5 ARG  CG      28 LYS  CB
31 ILE  CG1     28 LYS  CB
32 ILE  CG1     28 LYS  CB

24 VAL  CG1     27 ASN  CB
24 VAL  CG1     31 ILE  CB

 5 ARG  CG      28 LYS  CG
31 ILE  CG1     28 LYS  CG
32 ILE  CG1     28 LYS  CG

19 PHE  CB      34 LEU  CG

15 GLN  CB      27 ASN  CB
15 GLN  CB      31 ILE  CB
20 PHE  CB      27 ASN  CB
20 PHE  CB      31 ILE  CB

17 LEU  CD2     37 GLY  CA
34 LEU  CD2     37 GLY  CA

34 LEU  CG      37 GLY  CA

15 GLN  CG      37 GLY  CA
16 LYS  CD      37 GLY  CA
17 LEU  CG      37 GLY  CA

 3 GLU  CB      29 GLY  CA
 3 GLU  CB      38 GLY  CA

 3 GLU  CA      15 GLN  CG
 3 GLU  CA      28 LYS  CD
```

```
12 VAL  CA      15 GLN  CG
12 VAL  CA      28 LYS  CD
39 VAL  CA      15 GLN  CG
39 VAL  CA      28 LYS  CD

 3 GLU  CA      28 LYS  CG

 8 SER  CB      11 GLU  CG
26 SER  CB      11 GLU  CG

13 HIS  CA      16 LYS  CB
13 HIS  CA      24 VAL  CB
13 HIS  CA      39 VAL  CB

 4 PHE  CB      35 MET  CB
28 LYS  CE      35 MET  CB
32 ILE  CB      35 MET  CB

24 VAL  CB      27 ASN  CB
24 VAL  CB      31 ILE  CB
39 VAL  CB      27 ASN  CB
39 VAL  CB      31 ILE  CB

24 VAL  CB      27 ASN  CB
24 VAL  CB      31 ILE  CB

 4 PHE  CB      35 MET  CB
28 LYS  CE      35 MET  CB
32 ILE  CB      35 MET  CB

19 PHE  CB      34 LEU  CG

15 GLN  CB      27 ASN  CB
20 PHE  CB      27 ASN  CB
15 GLN  CB      31 ILE  CB
20 PHE  CB      31 ILE  CB

31 ILE  CG1     35 MET  CB
32 ILE  CG1     35 MET  CB

32 ILE  CD1     35 MET  CB

32 ILE  CG2     35 MET  CB
```

```
15 GLN  CG      37 GLY  CA
16 LYS  CD      37 GLY  CA
17 LEU  CG      37 GLY  CA

17 LEU  CD2     37 GLY  CA
34 LEU  CD2     37 GLY  CA

13 HIS  CA      17 LEU  CD1
13 HIS  CA      39 VAL  CG1

 3 GLU  CB      29 GLY  CA
 3 GLU  CB      38 GLY  CA

17 LEU  CB      34 LEU  CG

17 LEU  CB      34 LEU  CG

29 GLY  CA      35 MET  CB
38 GLY  CA      35 MET  CB

18 VAL  CA      21 ALA  CA
18 VAL  CA      25 GLY  CA
18 VAL  CA      33 GLY  CA

 3 GLU  CA      29 GLY  CA
 3 GLU  CA      38 GLY  CA

 2 ALA  CA      29 GLY  CA
 2 ALA  CA      38 GLY  CA

12 VAL  CA      15 GLN  CG
12 VAL  CA      27 LYS  CD
39 VAL  CA      15 GLN  CG
39 VAL  CA      28 LYS  CD

32 ILE  CG2     35 MET  CA

18 VAL  CA      21 ALA  CB
18 VAL  CA      24 VAL  CG1

 3 GLU  CA      28 LYS  CG

 3 GLU  CA      15 GLN  CG
 3 GLU  CA      28 LYS  CD
```

B

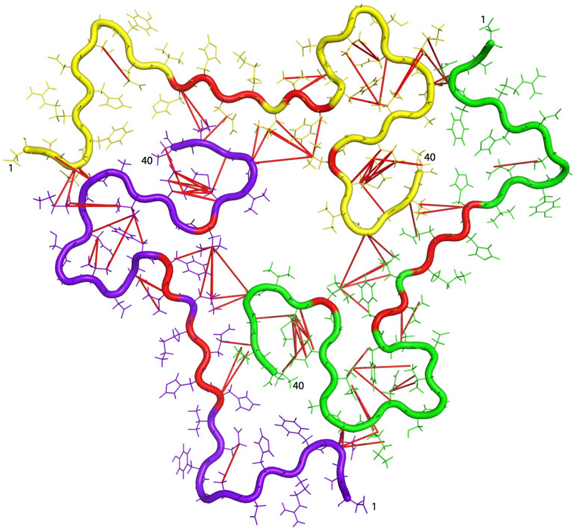

Fig. S9

All calculations discussed above assumed two monomers per fibrillar layer based on the data obtained by STEM. Here we explore the possibility of a trimeric structure, even if the STEM data speak against it. The set of unambiguous manual restraints listed in Table S1 could also be fulfilled by a trimer, in which the monomer assumes a very similar conformation as in the dimer. However, based on the set of upls with low ambiguity listed in (**A**), the possibility of a trimeric structure could be excluded. upls listed in a block of lines in (**A**) denote ambiguous restraints. While these restraints can be almost perfectly fulfilled for the dimer (target function 0.82), they lead to many violations in the trimer (target function 30.4). The best trimer structure is given in (**B**) with red backbone stretches indicating violations of TALOS backbone torsion angle restraints and red lines indicating violations of distance restraints. In agreement with the STEM analysis, the Osaka mutant fibrils are dimeric in our samples. This result was confirmed by automatic structure calculations including the full information from all NMR spectra, which yielded target functions of 16.4 (2.7/monomer) versus 78.6 (8.7/monomer) for dimer and trimer, respectively. These data confirm the formation of the fibril by two monomers per layer and the structure shown in Fig. 4.

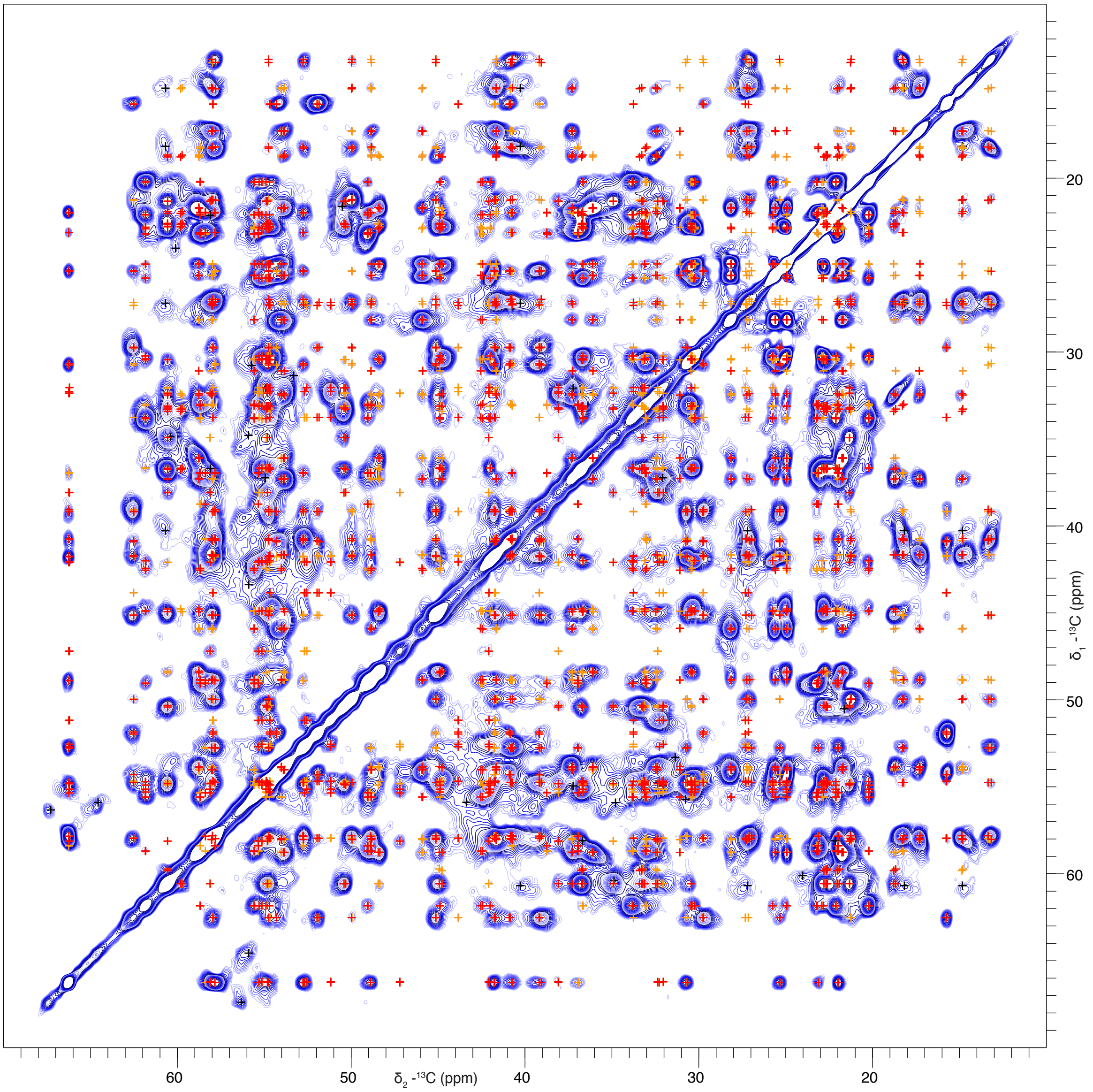

Fig. S10

Backcalculation of PAR restraint spectrum. Blue contours: aliphatic region of a PAR spectrum with 8 ms mixing time recorded on uniformly [^{13}C] labeled fibrils at 19 kHz MAS at a static magnetic field of 20.0 T (850 MHz proton frequency). Contacts between ^{13}C nuclei up to 6.5 Å were calculated from the structure of Fig.4 and back-predicted onto the spectrum. Red crosses mark the expected peak positions. Contacts between 6.5 and 7.5 Å are marked in yellow. Black crosses mark remainders of a second polymorph. The back-calculation matches the experimental spectrum.

.

DARR[a]	ω_1	ω_2
**E3-K28**	E3 CA	K28 CG
	K28 CG	E3 CA
	E3 CA	K28 CD
**E3-G29**	G29 C	E3 CA
**E3-A30**	E3 CA	A30 CA
	A30 CA	E3 CA
**H13-V40**	H13 CE1	V40 CA
	V40 CA	H13 CE1
	V40 C	H13 CA
	V40 C	H13 CD2
	V40 C	H13 CE1
	V40 CA	H13 CD2
**H6-E11**	H6 CD2	E11 CG
	E11 CG	H6 CE1
**F19-L34**	F19 CB	L34 CG
	L34 CG	F19 CA
	L34 CG	F19 CZ
**F20-I31**	F20 CG	I31 CG2
	F20 CE1	I31 CG2
**I32-M35**	I32 CG2	M35 CB
	M35 CB	I32 CG2
**M35-G38**	M35 CA	G38 C
	G38 C	M35 CA
	G38 C	M35 CE

PAR[b]	ω_1	ω_2
**E3-K28**	E3 CA	K28 CG
	K28 CG	E3 CA
**E3-A30**	E3 CA	A30 CA
	A30 CA	E3 CA
**H6-H13**	H6 CE1	H13 CE1
	H13 CE1	H6 CE1
**F19-L34**	L34 CG	F19 CA
	L34 CG	F19 CB
**I32-M35**	I32 CG2	M35 CB
	M35 CB	I32 CG2
	I32 CD1	M35 CB
	M35 CB	I32 CD1

CHHC[c]	ω_1	ω_2
**E3-K28**	E3 HA	K28 QG
**H6-H13**	H6 HE1	H13 HE1
	H13 HE1	H6 HE1
**F19-L34**	F19 QB	L34 HG
**I32-M35**	I32 QG2	M35 QB
	M35 QB	I32 QG2
	M35 QB	I32 QD1

PAIN[d]	ω_1	ω_2
**E3-G29**	E3 CA	G29 N
**H13-V40**	V40 C	H13 ND1
	V40 CA	H13 ND1
**Q15-G37**	G37 CA	Q15 NE2
**Q15-G38**	G38 C	Q15 NE2

PDSD[e]	ω_2	ω_1
**A2-A30**	A2 CA	A30 CA
	A30 CA	A2 CA
**E3-A30**	A30 CA	E3 CB
**H13-V40**	H13 CA	V40 CA
	V40 CA	H13 CA
	H13 CG	V40 CA
	V40 CA	H13 CG
	H13 CD2	V40 CA
	V40 CA	H13 CD2
**L17-L34**	L17 C	L34 CG
**L17-V36**	L17 C	V36 CB
**V18-L34**	V18 CA	L34 CB
	V18 CA	L34 CG
	L34 CG	V18 CA
**F19-L34**	F19 CA	L34 CB
	L34 CB	F19 CA
	F19 CA	L34 CG
	L34 CG	F19 CA
	F19 CG	L34 CG
**L34-G37**	L34 CG	G37 CA
	G37 CA	L34 CB

Table S1.

Unambiguous restraints extracted from the different spectra. Intermolecular restraints are colored red, intramolecular ones grey. No color indicates that the nature of the restraints is ambiguous with respect to their classification as inter- or intramolecular. (a) Restraints from 400 ms DARR spectra. To determine their inter- or intramolecular nature, the intensity of signals in uniformly labeled and diluted samples was compared. (b) Restraints from 8 ms PAR spectra. To determine their inter- or intramolecular nature, the intensity of signals in uniformly labeled and diluted samples was compared. (c) Restraints from 500 µs CHHC spectra. To determine their inter- or intramolecular nature, the intensity of signals in uniformly labeled and diluted samples was compared. (d) Restraints from 6 ms PAIN spectra. All contacts observed in this spectrum are intermolecular. (e) Restraints from 4 s PDSD spectra. No dilution analysis was done on this sample, and the restraints were not used in the manual structure calculation but are all confirmed in both manual and automated structures.

**NMR distance and dihedral constraints**	
Distance constraints (per monomer)	
Total PAR, PAIN, CHHC, PDSD	
Intra-residue	51
Inter-residue	
Sequential (li-jl = 1)	265
Medium-range (li-jl < 4)	359
Long-range (li-j > 5)	108
Intermolecular	164
Hydrogen bonds	19
Total dihedral angle restraints	
phi	28
psi	28
**Structure statistics**	
Violations (mean and s.d.)	
Distance constraints (Å)	0.01± 0.0001
Dihedral angle constraints (°)	0.46 ± 0.03
Max. dihedral angle violation (°)	3.4
Max. distance constraint violation (Å)	0.50
Deviations from idealized geometry	
Bond lengths (Å)	0.007
Bond angles (°)	0.6 °
Average pairwise r.m.s.d (Å)	
Heavy	0.56
Backbone	0.19
**Peak assignment statistics**	

Type of assignment	Type of restraint	DARR 8 ms U-[^{13}C]	CHHC 500 us U-[^{13}C]	PAR 8 ms U-[^{13}C]	PDSD 4 s [2-^{13}C-glc]	PAIN 6 ms U-[^{15}N,^{13}C] 50/130 ppm	total
	Tolerance (ppm)	-	0.2	0.2	0.2	0.2	
	Upper distance limit	7.0 Å	5.5 Å (^{1}H-^{1}H)	6.5 Å	8.0 Å	5.5 Å	
CYANA assignment	Intra-residue (1 ≤ li – jl)	-	484	442	138	74/90	1228
	Sequential (1 ≤ li – jl)	-	591	543	264	73/84	1555
	Medium-range (2 ≤ li – jl ≤ 4)	-	352	388	238	23/17	1018
	Long-range (5 ≤ li – jl)	-	101	84	66	0/0	251
	Intermolecular	-	399	368	284	22/14	1087
Manual analysis	Intramolecular	12	6	8	11	0	37
	Intermolecular	12	1	4	10	5	32

Table S2.

NMR, structure and peak assignment statistics. The pairwise r.m.s.d. was calculated among 10 refined structures of a single bundle. The r.m.s.d. of a bundle of twenty independent bundles was 1.06 Å (heavy atom) and 0.80 Å (backbone). Whereas CYANA assignments from the final cycle are not necessarily spectrally ambiguous, manually assigned peaks are unambiguous in both dimensions and were classified as intra- or inter-monomer based on dilution analysis and the use of mixed labeled samples. The DARR spectrum was not used for automatic structure calculation. The PDSD restraints were not entered into the manual calculation shown in Figure 3 as their inter/intra-molecular nature could not be determined from the NMR data a priori.

Exp.	Isotope labeling	Mixing [ms]	CP [ms]	RF amplitude for mixing [kHz]	Acquisition t_{1max}x t_{2may} [ms]	d_1 [s]	Exp. time [h]
DARR	uniform [^{13}C]	400	1.0	19 (^{1}H)	10.2 x 15.4	2.3	25
DARR	dilute [^{13}C]	400	1.0	19 (^{1}H)	10.2 x 15.4	2.5	211
PAR	uniform [^{13}C]	8	0.8	66 (^{13}C), 63(^{1}H)	10.2 x 15.4	2.5	57
PAR	dilute [^{15}N,^{13}C] / NA 1/4	8	0.8	66 (^{13}C), 63(^{1}H)	10.2 x 15.4	2.5	205
CHHC	uniform [^{13}C]	0.5	1.0, 0.2, 0.2	-	10.2 x 15.4	2.5	34
CHHC	dilute [^{15}N,^{13}C] / NA 1/4	0.5	1.0, 0.2, 0.2	-	10.2 x 15.4	2.5	205
PAIN	uniform [^{15}N,^{13}C]	6	1.4	45(^{15}N), 46 (^{13}C), 71(^{1}H) (carrier at 50 ppm)	10.2 x 15.4	2.5	23
PAIN	uniform [^{15}N,^{13}C]	6	1.4	43(^{15}N), 40 (^{13}C), 68(^{1}H) (carrier at 130 ppm)	10.2 x 15.4	2.5	23
PAIN	mixed [^{15}N]/[^{13}C] as 1/1	6	1.4	43(^{15}N), 43 (^{13}C), 67(^{1}H) (carrier at 50 ppm)	7.7 x 12.9	2.2	23
PAIN	mixed [^{15}N]/[^{13}C] as 1/1	6	1.4	43(^{15}N), 42 (^{13}C), 70(^{1}H) (carrier at 130 ppm)	7.7 x 12.9	2.2	38
PDSD	sparse [^{15}N,1-^{13}C glucose]	4000	1.0	-	12.8 x 18.0	1.3	181

Table S3.

NMR experimental parameters. All spectra were acquired at a static magnetic field of 20.0 T (proton frequency 850 MHz) at samples temperature of 5-15 ˚C. MAS frequencies were 19 kHz for all experiments except for the PDSD, which was acquired at 11.5 kHz to favor spin-diffusion. Decoupling of ^{1}H during acquisition was achieved using SPINAL 64 at 100 kHz.
